# Embedding Physical Activity into Community-Based Peer Support Groups for those Severely Affected by Mental Illness

**DOI:** 10.3390/ijerph20032291

**Published:** 2023-01-27

**Authors:** Laura C. Healy, Adam Benkwitz, Zoe McVinnie, Mustafa Sarkar, Mel Islin, Andy Brinded, B. Dodge, Sofija Opacic, Zoe Swithenbank, Shanika Ranasinghe, Jennie Oliver, Maria Karanika-Murray, Mary E. Nevill

**Affiliations:** 1 Sport, Health and Performance Enhancement (SHAPE) Research Centre, School of Science and Technology, Nottingham Trent University, Nottingham NG11 8NS, UK; 2 Sport, Physical Activity and Health Research Centre, Newman University, Birmingham B32 3NT, UK; 3 Department of Psychology, Glasgow Caledonian University, Cowcaddens Road, Glasgow G4 0BA, UK; 4 Rethink Mental Illness, The Dumont, 28 Albert Embankment, London SE1 7GR, UK; 5 School of Business, University of Leicester, Brookfield, 266 London Road, Leicester LE2 1RQ, UK

**Keywords:** severe mental illness, peer support, physical activity, mental health, community

## Abstract

Despite a growing evidence base on the effectiveness of community-based physical activity interventions for mental health, there is a lack of studies that focus on those affected by severe mental illness (SMI), who often experience poorer physical health, and are less physically active than the wider population. The use of peer support groups in this context is also understudied, despite benefits being documented in other contexts. This study examined the impact and process of a nationwide project to embed physical activity into peer support groups for those affected by SMI. Following the embedding of physical activity within peer support groups, interviews and focus groups were conducted to explore the experiences of those involved with the project and analysed using reflexive thematic analysis. The key findings related to: 1) the social aspects of embedding physical activity in the groups; 2) the focus on peer support and informal physical activity (rather than organised sport) being beneficial; 3) doing things differently and lessons to learn; and 4) the impact of the COVID-19 pandemic. Overall, we found that peer support is an important feature to include in projects encouraging those severely affected by mental illness to become more physically active.

## 1. Introduction

There is a growing body of literature on mental health and physical activity that recognises the importance of the settings, contexts, and cultures where activities take place [1], as these activities have varying ‘ingredients’ and may have varying outcomes [2]. Research has considered the relationship between mental health and physical activity across contexts such as (but not limited to): community settings [3,4], medium secure mental health settings [5,6,7], football clubs [8,9], and other community sport settings [10,11]. Increased physical activity for individuals affected by poor mental health has been associated with a range of physical health benefits, such as improved cardiovascular fitness, lower blood glucose, and lower body fat percentages [12]. Furthermore, research has shown a wide range of psychosocial benefits. For example, physical activity interventions for those with poorer mental health have been shown to mitigate the negative impact of social isolation [13,14]. Physical activity interventions can provide a space to interact with other people and to rehabilitate social skills [1], as well as creating a social identity that encourages physical activity engagement [15]. 

Initiatives promoting physical activity for mental health have also been identified as contributing to mental health recovery. For example, studies examining community football mental health programmes have shown that such initiatives can contribute to personal recovery [14]. Other research has shown that physical activity promotes personal recovery through the processes identified in the CHIME framework [16]. Specifically, physical activity initiatives can support mental health recovery through connectedness (peer support, developing relationships, being part of a community, shared experiences), hope and optimism about the future (seeing beyond their current circumstances and feeling more positive about health and recovery), developing or regaining identity (regaining a sense of self separate to mental illness, benefits to social identity), provide meaningful activities (physical activity being personally important to participants in relation to health and recovery), and empowerment (volition in choosing whether to attend, opportunities for decision-making within sessions) [5]. Physical activity interventions can also impact on social factors, such as rebuilding a social identity and being involved in active citizenship (contributing to the wider community), as well as shared decision-making and co-production (being involved in decisions about how activities are delivered) [8,17].

Despite the evidence relating to community-based physical activity interventions for mental health (i.e., community members and leaders from a range of settings working together to promote physical activity [18]), there remains a scarcity of focus on those severely affected by mental illness (SMI), which is problematic as these individuals often experience poorer physical health [7,19], are less physically active, and are more sedentary than the wider population [20]. In the United Kingdom, the death rate for people affected by SMI is two to three times higher than that of the general population [21], a large proportion of which is attributable to non-communicable illnesses that may be prevented or made less severe through regular physical activity. Of the few studies that have examined physical activity within those affected by SMI, there have been positive benefits including improved social interaction and physical and mental health [22]. However, barriers have also been identified. For instance, a systematic review and meta-analysis of barriers towards exercise in those affected by SMI identified that while psychological barriers such as stress and depression were commonplace, there were also socio-ecological barriers that include the lack of support, including peer support [23]. Research in this area has often been conducted on a small scale, therefore there is a need to examine larger scale projects where community physical activity initiatives are delivered nationwide, while still appreciating the local context [1]. Additionally, research is needed to explore the role of peer support for mental health within community-based physical activity interventions [1], particularly to appreciate the challenges, as well as the positive outcomes, of engaging and treating individuals in the ‘real world’ [24]. The present study addresses this need, by examining a project delivered by a national charity which aimed to embed physical activity into peer support groups for those affected by SMI.

Rethink Mental Illness are an England-based charity supporting everyone affected by SMI to have a good quality of life, which includes supporting both mental and physical health. Key to their provision are peer support groups; a network of over 140 local support groups offering ‘a welcoming, non-judgmental space to talk about your experience and get peer support’ [25]. These groups are open to anyone affected by SMI, including carers and family members. Support from peers and peer leaders has previously been found to be a key ingredient in the adoption and adherence stages of physical activity interventions [26]. In some contexts (especially U.K. mental health services), the term “peer support” can mean a specific (sometimes paid) role using their lived experiences of mental illness to support others [27]. It can also mean peers supporting each other as a group, often with a designated peer leader; the latter is how Rethink Mental Illness’s groups operate. 

Rethink Mental Illness was funded by Sport England to address some of the barriers preventing people affected by SMI from engaging in physical activity by embedding activities into peer support groups and local services. The aim was to support individuals who are currently inactive to engage in physical activity for at least 30 min per week, which could be split into 10-min chunks if that was more suitable based on individual needs. Groups were encouraged to embed the physical activity over a 12-month period. During the 3-year intervention, the target was to engage and support forty-six groups across England to start facilitating physical activity: six in Year 1, and twenty in each of Years 2 and 3. Using a co-produced toolkit and personalised guidance provided by the project managers, peer support groups and services were supported to start facilitating physical activity opportunities, based on group members and service users’ preferences. The level of support provided to groups was based on need – a combination of training, equipment, signposting, and advice. A wide range of physical activities were adopted by groups, examples of which include running, yoga, walking, badminton, chair-based exercises, and some groups who delivered a variety of different activities over the course of the 12-month period.

The aims of our evaluation were to: 1) investigate the impact of peer support and social interaction on physical activity behaviours, and physical and mental health for individuals affected by SMI; 2) understand if there were any benefits from this for those affected by SMI; and 3) understand the processes facilitating or hindering project delivery. 

## 2. Materials and Methods

### 2.1. Methodology

While the overall evaluation adopted a mixed-methods approach, for the purposes of this paper, we are only reporting on the qualitative elements of the project. As we were interested in how the structural conditions and social-cultural contexts of the project impacted on the participants’ experiences, we underpinned our approach in a relative ontology (assuming numerous subjective realities) and a constructionist epistemology (our understanding is based on appreciating multiple social constructions of knowledge). This approach has been adopted in prior research investigating physical activity in mental health contexts, including studies focusing on populations affected by SMI [5,8]. This approach also appreciated the complexity of the nationwide project, with different groups of diverse membership and needs. This also led us to employ semi-structured individual and focus groups interviews as our primary form of qualitative data collection, to value and explore participants’ experiences [28] of the project. Both methods have previously been successfully used in mental health research [1,29].

Interviews were conducted with peer support Group Members, Group Leads (responsible for coordinating groups and sometimes delivering the physical activity), and Group Development Officers (GDOs) working across different geographical regions, to holistically help peer support groups. GDOs were heavily involved in recruiting groups to participate in the project. Finally, interviews with the Project Managers of the physical activity project were undertaken at the end of the process. Ethical approval was provided by the Non-invasive Human Ethics committee at the first author’s institution (reference: 18/19-52).

Aligned with the values of Rethink Mental Illness, we involved individuals with lived experience of being affected by SMI in the evaluation process. Peer researchers were recruited through Rethink Mental Illness’s networks and trained by the research team. Training consisted of two face-to-face workshops on the purpose of the evaluation, ethical research practice, facilitating data collection, and data analysis. This role involved supporting the research team when visiting groups for data collection, as well as analysing and interpreting the data. Peer researchers were valued members of the evaluation team, were paid for their contributions to the evaluation, and are named as co-authors on this paper (authors 6 –11).

### 2.2. Participants and Procedure

Data from 26 interviews are presented here: 5 focus groups with peer support group members (28 participants in total, M = 5.6 participants, range = 3–11 participants, three groups in South-East England, 1 in London, and 1 in South-West England), 16 individual interviews and 2 focus groups (7 participants in total, 3 participants in one focus group and 4 in the other) with group leads (leading groups in London, South-East England, South-West England, East Midlands, East England, and North-East England), 1 focus group with GDOs (3 participants), and individual interviews with the 2 project managers. All participants provided informed consent to take part in the research and did so voluntarily; no compensation was offered to any participants. The focus groups with peer support group members were conducted in person, usually in the setting where the group regularly met in order to facilitate an environment where participants felt relaxed and comfortable. They were facilitated by one of authors 1–4 who were educated to doctoral level with extensive experience in conducting qualitative research, and in most cases were supported by peer researchers who had received training in qualitative research methods and brought their own lived experience of being affected by SMI. The focus groups and interviews with group leaders, GDOs, and project managers were predominantly remotely conducted using phone calls or online video conferencing software, although one group lead interview was conducted in person due to ease of access to the participant. Even prior to the COVID-19 pandemic, the use of virtual technology for interviews in a mental health context was identified as being beneficial to reach a geographical spread of participants more safely, cheaply, and quickly than face-to-face meetings [30]. The focus groups and interviews with group leads were conducted by one of authors 1–4. Regardless of how they were conducted, all focus groups and interviews were recorded before being transcribed verbatim. 

The interviews and focus groups were semi-structured, which allowed for key topics to be discussed with all participants, while also allowing the conversation to move to subjects deemed important by those being interviewed. Interview guides were developed based on the key literature in the area [8,11,16,17] and the overall aims of the evaluation. For the focus groups for peer support group members, questions focused on the broad themes of their experience of being involved with the peer support group, their views on physical activity (including their views on the physical activity embedded in their support group), and their mental and physical health. Interviews and focus groups with group leaders covered areas including their background and how they became involved with the peer support groups, their own views on physical activity, their experience of embedding physical activity into peer support groups, and their views on the toolkit created by Rethink Mental Illness. Interviews and focus groups with the GDOs and project managers focused on their experiences of delivering the project, including the recruitment of groups to be involved, their perceptions of how embedding physical activity impacted group members and group leads, and the facilitators and barriers of project delivery. The focus groups with peer group support members took place between May 2019 and February 2020, approximately three months after the group had started embedding physical activity into their sessions. No focus groups were conducted with groups following this point due to the COVID-19 pandemic, which impacted both the ability of the groups to meet and the access of the research team to group members. Interviews with the group leaders, GDOs, and project managers took place between May 2019 and August 2021. As such, in interviews following the onset of the COVID-19 pandemic (circa March 2020), participants were asked questions relating to the impact of the pandemic on group members and the delivery of the project, Supplementary Materials: File S1.

### 2.3. Data Analysis

Data were thematically analysed, aligned with the reflexive approach outlined by Braun and Clarke [31], as themes “were generated by the researcher[s] through data engagement mediated by all that they [brought]” to the process, and also that “the process was unstructured and organic, with the potential for codes to evolve to capture the researcher’s deepening understanding of the data” (p. 39). Data were inductively analysed, with consideration to our original aims and the previous literature, but without using these aspects as a specific framework as a lens through which to view the data. Authors 1-4 individually analysed transcripts, creating codes from the raw and developing initial themes. These initial themes, with supporting quotes, were then discussed in group meetings with the entire research team (including peer researchers) held over the project duration used to discuss and reflect on the themes, and consider the interpretations and language involved, before refining and writing up [32,33]. The process ensured the analysis was rigorous through ‘critical friends’; the research team engaged in reflective conversations to challenge and develop the themes generated. These discussions were not to achieve consensus, but to ensure the data had been fully interrogated, to produce a robust analysis [34].

## 3. Results

The analysis revealed that peer support is very important for those involved, and that peer support groups are both successful at promoting physical activity and valued by those affected by SMI. Four key themes identified were: 1) social aspects of embedding physical activity in groups; 2) focus on peer support and informal physical activity (rather than organised sport) was beneficial; 3) doing things differently and lessons to learn; and 4) the impact of the COVID-19 pandemic. 

### 3.1. The Social Aspects of Embedding the Physical Activity in Peer Support Groups

The social aspects of the groups were an important part of the project. Both group members and group leads acknowledged this was at least as beneficial and valued as the physical activity itself:

“The social side, groups and things, the social side here is so important, it’s a very, very important side of it… because you trust each other and have a bit of banter and a bit of fun, and it just feels so at home; so free, so easy. You come in and you talk to anyone about anything you want, and you know it won’t go any further, which is very important for a lot of things.” (Peer support group member) 

“It was hugely important. I know a lot of the people who were doing the activities said, ‘There’s no way they would’ve kept it going on their own’. Having the group, having the chat, having the encouragement from each other was really key for them.” (Group lead) 

Participants identified many benefits that were facilitated and encouraged by the peer support groups. One group member acknowledged how they felt about joining in the group activities: “It’s very uplifting so when I start, I don’t feel, for example, energetic, but when we join in a group these physical activities give me energy and positivity and I feel better in myself”. Further benefits were identified by a group lead: 

“Yeah the one really big take away positive thing is that it was so hugely beneficial to our carers. Absolutely mentally and physically, emotionally, we could connect with them, they could connect with us and they could connect with each other. So it ticks so many boxes, broke down social isolation, provided peer support, provided group support, gave access to the staff, all through walking and physical activity. It was brilliant. The main thing is it might not sound very much to a lot of people but just to have a coffee bought for you that’s incredible.” (Group lead)

Group members also reported feeling more comfortable in social situations: 

“Oh yes, all the tai chi and that, that’s a great help. I always feel when I come in and when I leave here, I feel a much better person… I can go into a crowd now, I’m happy. Before I came to Rethink, I could never talk with a group like this, I’d probably just sit back, I know it’s done a lot for me.” (Peer support group member)

### 3.2. Focus on Peer Support and Informal Physical Activity, Rather Than Organised Sport, Was Beneficial

There was diversity in the types and frequency of physical activity adopted by groups. A focus on peer support and informal physical activity was considered better by participants than organised sport. This was partly because of the nature of the activities themselves, with walking being popular with group members: “I go for walks, coming here, walking to the local shops, something like, I enjoy walking a lot, ‘cause it’s fresh air and there’s sunlight and I like that a lot”. Activities such as walking were also favoured, as it was an activity that could be easily picked back up if a group member could not attend every week:

“Walk and talk sounds really good because it means getting out, it means social interaction both of which are major achievements but it’s not got that large challenge of having to have a level of physical fitness to achieve it. It’s something that you can attend then miss a few weeks and then come back to.” (Peer support group member) 

Group leads also found planning organised sport activities challenging due to not knowing how many group members might attend a specific session.

“Say if we have things set up for rounders, you have to have a set number of people to be able to play the game and if there’s only a couple of people turn up, you can’t play the game so it then turns into something else okay? … it’s like trying to choose some sort of activity that doesn’t take up massive numbers and … if only a few people turn up then we can still…still work with it.” (Group lead)

Group leads were highly significant to the success of the project, as trusted members of groups with an excellent understanding of their specific needs and contexts. 

“I need to pay more attention to what people want rather than just planning and expecting them to fit in with what I think will work well. I need to go to them first and do some more insight work into what they want rather than just planning and do it the other way around a bit. Because especially with groups of people that have got mental health problems, like [name of participant] said, it’s not going to be everybody’s going to turn up every week and follows this lovely path, life isn’t like that. So, yes, I’ve learnt to talk to groups first and then plan afterwards.” (Group lead)

Despite the value placed by Rethink Mental Illness on co-design and co-production, some group leads and GDOs felt that those delivering the project could have been more involved in discussions from the conception. For instance, the GDOs felt that the target for the number of groups to be engaged within the project was overly ambitious: 

“I think that’s the biggest learning was that KPIs that were set were wholly unrealistic… the KPIs were 46 peer support groups in total. When in total we had 140 across the organisation. So to expect a third to want to be on board with it when some of them running for 10/20 years, and very, very set in their ways, and very happy with the way that they were operating. And I’m not sure how much consultation there was done.” (Group Development Officer) 

The GDOs also reported challenges in recruiting existing peer support groups to embed physical activity into their meetings. 

“The thing that really has struck me more than anything else is when you’re aiming something at people to help them with their mental health and especially those who have challenges in that area, I think you have to be very careful about the level of expectation you put on people. For some people actually getting up is a big success for the day, that’s a major achievement, getting showered as well, huge step forward. So regularly attending an event I think is a big ask.” (Group Development Officer)

The points highlighted here relating to groups of people affected by SMI are important to consider for future intervention work, and thorough co-production involving those ‘on the ground’ is deemed to be the most suitable way to plan activities that are going to be beneficial at a local level.

### 3.3. Doing Things Differently, Lessons to Learn

Participants suggested changes to make similar projects more effective. These included altering timescales and project planning, using resources such as toolkits to support group leads, and the sustainability of funding for practicalities. Data also emphasised recognising the diversity of groups in their local contexts, and partnering with other organisations, as beneficial approaches. It was felt that key elements could have been changed in the planning to facilitate successful delivery of the project, as identified by a GDO: “The takeaway was the groups were actually quite engaged and ready to try and do things, but we just didn’t quite have the tools and the collaboration within the organisation to get that actually working”. Another suggestion related to having a longer pre-delivery period (i.e., before groups started embedding physical activity), which would have allowed greater co-production at group level, and for enthusiasm for the project to be generated: 

“The timescales that were set weren’t realistic, in terms of actual delivery. So again, this is probably one of the biggest pieces of learning that we found, and that I found… you know, there was a three-year project and three years of delivery, where in hindsight there probably should have been at least a six month initiation phase before you start delivery. It’s impossible to start delivery from day one because nothing was set up prior to that. That was a huge challenge to then work out how can we fit in three years worth of delivery effectively within two/two and a half years, pandemic aside.” (Project Manager) 

“I think if we’d that better collaboration, improved collaboration in the beginning and understood that we’d have a better idea of how to approach it, how to roll it out and implement things.” (Group Development Officer) 

It was recognised that differences in groups and their local context meant a one-size-fits-all approach was inappropriate. With this diversity came challenges, both at a local level (such as meeting the needs of the whole group) and more widely in ensuring the physical activity on offer was appropriate for a range of different groups.

"I think Rethink is trying to acknowledge and provide the resources they have with the physical activities, equipment that was provided to the groups. I think that’s important to note, but I think because of the unique characteristics of this group, language, background, capabilities of whether people are able to, physical capabilities, mental health capabilities, it does complicate the situation in terms of how we can support the group best." (Group lead) 

However, there were also positives of having a diverse group of people coming together as peer support groups. 

"With our group, we had so many different people coming, so we’d have some people coming from school, like, with their carers, coming from college, coming from work, or that didn’t work, just coming from home, and just bringing so many people together that wouldn’t normally interact with each other." (Group lead) 

### 3.4. Impact of COVID-19 Pandemic

The pandemic had a profound impact on project delivery, and those involved in groups. Many groups ceased to meet – some altogether. While some groups were able to maintain contact using online services and communication apps, the lack of face-to-face contact had considerable impact. It further reinforced the importance of peer support for many individuals severely affected by mental illness.

“I believe that the pandemic has really literally changed the whole way of how we function and especially how groups are functioning. We’ve not been able to do WhatsApp or use online formats at this point because when asked collectively to the group members, “What is your preference to stay in touch?” Most of them don’t have the skills and they feel comfortable by just having that phone call to have that conversation about how they’re coping during the pandemic without the group meeting for face to face activities. So it’s hit them hard, and it’s hit me hard, because we’re all part of the group.”

(Group lead)

“I think they’ve realised that maybe we’ve taken for granted, but this has been a really good experiment in a sense, where we’ve recognised what really makes peer support, what makes that up, and that human presence and all the non-verbal cues and that human presence is what does contribute to being a supportive group face-to-face.”

(Group Development Officer)

Individuals’ experiences during the pandemic reinforced that, for those affected by SMI, groups could be a vital way of avoiding social isolation. Groups were perceived to be an important aspect in maintaining social connections, as well as promoting physical activity.

“I think having the group there is probably a good thing and I know one of the runners he stopped running over the pandemic period, but remained involved with the group just because he was getting the benefits of the social aspect of the group, which is really good. I think it’s all about isolation isn’t it.”

(Group lead)

“Obviously the exercise has got to be good for everyone, but I think especially with the pandemic and the isolation and that as a social group it’s really paid dividends and I think it’s a shame we didn’t get a walk and talk group up and running before the pandemic because I think that would’ve been a good social connection as well for people.”

(Group lead)

There was a clear sense that the pandemic resulted in reduced levels of physical activity for all involved, due to the impact of lockdown on individuals, but also the lack of contact with peer support groups that facilitated physical activity. However, for some individuals, there was a sense there had been a change in the importance of physical activity.

“Yes, it definitely dropped, definitely dropped during the pandemic and I think that was linked to the drop in social contact. I think for me with less of a face-to-face contact with people it dropped my natural energy levels and I found it quite hard to motivate myself to do things. I think getting back out now, starting to build those levels of contact I’m definitely seeing my motivation to do things pick up.”

(Group lead)

“I think as well, it’s gone to show how important physical activity is, so especially in the first month or so when the very toughest lockdown, when exercise was the one exemption out of your house, other than shopping really, I think that showed how important it is to be grateful for what you can do, and no matter your disability or not, then exercise is just so important, physically and mentally. So I definitely think that’s highlighted the importance of this group specifically.”

(Group lead)

Additionally, the pandemic made communication across different levels of the project (i.e., project managers, GDOs, group leaders) challenging. For example:

“Well it was really the pandemic because it’s that face to face contact that was a problem. So it was the digital isolation, the digital exclusion and in terms of cost and finances but also in terms of intelligence, know-how and training. So that hindered some people, yes, because not everybody’s able to access online services and if they are able to they need an awful lot of support to do that. Rethink did make some support available and there is support out there but it’s still difficult for some people.”

(Group lead)

“There’s a huge number of members in our groups and the coordinators that don’t have access to technology. And there’s a number of barriers, whether that be affordability, confidence in using it, or just through their own wellness, aspects of their being unwell that they won’t use it. And we’ve got a lot in areas where it’s really difficult, particularly in a lot of rural areas where you’ve stuck to only using one provider and it not being great.”

(Group Development Officer)

## 4. Discussion

The aims of the present study were to examine the impact of embedding physical activity into peer support groups for individuals affected by SMI, delivered by a national charity. We also examined the processes which facilitated and hindered project delivery. Our findings reinforce that the social aspect of community-based activities is highly attractive to mental health service users [1] and may be more appealing than the activity itself [35]. Additionally, an understanding of local contexts, and recognising the importance of key individuals such as group leaders, was important for successfully embedding physical activity in support groups. On the other hand, our findings suggest that organisations that are planning on delivering similar projects in relation to physical activity and peer support for those affected by SMI need to consider whether timescales for implementation are realistic, as well as engaging key individuals “on the ground” throughout project design and delivery to ensure that it is meeting the needs of the groups.

Our study adds to the limited evidence regarding the role of peer support in community initiatives aimed at promoting physical activity for mental health for individuals affected by SMI. By focusing on the experiences of the individuals affected by SMI - who are at particular risk of being less physically active and experiencing poor physical health outcomes - we have addressed an important gap in the literature. The social element of peer support groups was reported as vital and highly valued by those involved, aligning with the limited existing evidence in this specific area [1]. This is supported by the recovery literature that highlights key processes essential to supporting individuals with mental illness in their recovery [36], with ‘connectedness’ being a central element [16]. Social interactions in the groups appear key in enabling engagement with being physically active [23], with peer group leaders being crucial to facilitate the success of groups. Support is needed for the group leads’ own development, to ensure that they have the confidence and skills to facilitate physical activity sessions (or can enlist help to do so) and support individuals’ personal and social recovery [8,17] in the community. The data suggested that more consideration should be given to what constitutes physical activity for those affected by SMI, as informal activities seem to be preferred compared to formalised ‘sport’ by some groups. However, given that another key part of the recovery processes is to support individuals with activities or interventions that they find ‘meaningful’ [16], engaging at a local level (even within a national project) to learn what people might find meaningful, and the (sub)culture and language relevant to the local groups [11], can aid engagement. Co-production between group members, group leads, GDOs, and management (or equivalents) is vital across all aspects of projects, recognising local contexts and variations in the needs of groups. 

Regarding practicalities, participants across the project reported that the timescales for projects need to be realistic, with adequate time prior to groups beginning activities. This allows more time for co-production, planning, and communication. Flexibility in expectations would also help group leaders to facilitate physical activity in their location and context. This has been previously identified as being helpful for mental health service users [37,38], as it helps mitigate barriers relating to feeling pressure or expectation [1].

### 4.1. Strengths and Limitations

There are several strengths of this evaluation approach. The combination of data collected via individual and focus groups, with group members, group leads, GDOs, and project managers, enabled an in-depth exploration of perceptions of the impact of the project and key outcomes, such as physical activity, mental health and wellbeing, and physical health, whilst valuing the participation and authenticity of individuals’ involvement [28]. An additional strength comes from the involvement of the peer researchers. The expertise they brought from their own lived experience of SMI was invaluable to the collection and interpretation of the data. Their contribution to the data analysis and particularly comments related to how the data ‘resonated’ [39] with them, offers confidence that these findings can be generalised to other projects conducted within similar contexts. 

There were also challenges that need to be acknowledged when considering the scope of the findings. A particular challenge was access to and the engagement of group members by the evaluation team. Data collection was mostly completed in support group sessions, access to which was almost exclusively facilitated through group leads. Direct access to individual group members may have allowed engagement with participants regardless of their attendance at support groups. This issue was exacerbated by the onset of the pandemic, as – without contacts for group members – attempts to collect data relied on information being passed on by groups leads. Despite the unique situation caused by the pandemic, the opportunities to maximise access to participants should be considered by teams conducting evaluations, as well as the organisations commissioning them, so the experiences of a wide range of individuals involved in projects can be captured. 

### 4.2. Future Directions and Recommendations

Our study addresses the need for more evidence for community-based peer support interventions using physical activity that can provide new and needed knowledge on the specific approach of using peer support groups with a peer group lead. Several recommendations emerge from our findings for organisations planning to use peer support groups to encourage people affected by SMI to be more active. Based on our evaluation, we propose the following four key principles for future work in this specific area. First, the social elements of peer support groups are highly valued and essential ingredients for positive outcomes. Second, it is important group leaders have confidence and experience in physical activity (or know where to gain support with this), as well as expertise in the needs of their group(s). Third, co-production should involve a wide range of stakeholders, from project conception to development, implementation, and evaluation. Finally, communication between the organisations running projects and those practically delivering them is crucial to their overall success. 

In relation to future research, it is as yet unclear as to whether physical activity, peer support, or the combination of the two is most important for promoting physical and mental health for those affected by SMI. Evidence from other contexts would suggest that social elements bring about greater benefits for long-term health and behaviour changes than the physical activity per se [40,41]; however, to the best of our knowledge, this has yet to be directly examined with a population affected by SMI. As such, studies comparing the impact of physical activity and peer support on their own, as well as in combination, would be a well-placed extension of the literature. That said, this may be hard to implement in a real-world community context, such as the setting of the present research, so careful consideration should be given to the design and implementation of such a study in order to take into account the views and preferences of participants.

## 5. Conclusions

This research highlights the value of peer support when supporting individuals affected by SMI to engage in physical activity. We provide key recommendations for both researchers and organisations involved in delivering physical activity and peer support projects for people affected by SMI. Embedding physical activity in peer support groups and engaging key stakeholders throughout this process can promote a range of benefits for those involved. 

## Data Availability

To maintain the confidentiality of the participants the research data is not available.

## Supplementary Materials

The following are available online at https://www.mdpi.com/article/10.3390/ijerph20032291/s1, File S1: Focus group and interview guide themes.

## References

[1] Tweed L.M., Rogers E.N., Kinnafick F.-E. (2020). Literature on Peer-Based Community Physical Activity Programmes for Mental Health Service Users: A Scoping Review. Health Psychol. Rev..

[2] Smith A., Jones J., Houghton L., Duffell T. (2016). A Political Spectator Sport or Policy Priority? A Review of Sport, Physical Activ-ity and Public Mental Health Policy. Int. J. Sport Policy Politics.

[3] Harrold S.A., Libet J., Pope C., Lauerer J.A., Johnson E., Edlund B.J. (2018). Increasing Physical Activity for Veterans in the Mental Health Intensive Case Management Program: A Community-Based Intervention. Perspect. Psychiatr. Care.

[4] Lesley M.L., Livingwood K. (2015). Assessing Sustainability of InSHAPE Participants’ Fitness Activities in a Community Mental Health Setting. J. Psychosoc. Nurs. Ment. Health Serv..

[5] Benkwitz A., Morris M., Healy L.C. (2019). An Ethnographic Study Exploring Football Sessions for Medium-Secure Mental Health Service-Users: Utilising the CHIME Conceptual Framework as an Evaluative Tool. J. Psychosoc. Rehabil. Ment. Health.

[6] Rogers E., Kinnafick F.-E., Papathomas A. (2019). Physical Activity in Secure Settings: A Scoping Review of Methods, Theory and Practise. Ment. Health Phys. Act..

[7] Rogers E., Papathomas A., Kinnafick F.-E. (2021). Inpatient Perspectives on Physical Activity in a Secure Mental Health Setting. Psychol. Sport Exerc..

[8] Benkwitz A., Healy L.C. (2019). ‘Think Football’: Exploring a Football for Mental Health Initiative Delivered in the Community through the Lens of Personal and Social Recovery. Ment. Health Phys. Act..

[9] Friedrich B., Mason O.J. (2017). Evaluation of the Coping Through Football Project: Physical Activity and Psychosocial Outcomes. Open Public Health J..

[10] Get Set to Go Programme Evaluation Summary 2014-2017. https://www.mind.org.uk/about-us/our-policy-work/sport-physical-activity-and-mental-health/resources/get-set-to-go-programme-evaluation-summary-2014-2017/.

[11] Wilcock R., Smith A., Haycock D. (2021). Designing Community Sports-Based Programmes for Men with Mental Illness: A Quali-tative Study of the Offload Rugby League Programme. Ment. Health Phys. Act..

[12] Kandola A.A., Osborn D.P.J. (2022). Physical Activity as an Intervention in Severe Mental Illness. BJPsych Adv..

[13] Taylor D., Pringle A. (2022). Investigating the Effect of Walking Football on the Mental and Social Wellbeing of Men. Soccer Soc..

[14] Magee J., Spaaij R., Jeanes R. (2015). “It’s Recovery United for Me”: Promises and Pitfalls of Football as Part of Mental Health Re-covery. Sociol. Sport J..

[15] Soundy A., Freeman P., Stubbs B., Probst M., Coffee P., Vancampfort D. (2014). The Transcending Benefits of Physical Activity for Individuals with Schizophrenia: A Systematic Review and Meta-Ethnography. Psychiatry Res..

[16] Leamy M., Bird V., Boutillier C.L., Williams J., Slade M. (2011). Conceptual Framework for Personal Recovery in Mental Health: Systematic Review and Narrative Synthesis. Br. J. Psychiatry..

[17] Ramon S. (2018). The Place of Social Recovery in Mental Health and Related Services. Int. J. Environ. Res. Public Health.

[18] Bopp M., Fallon E. (2008). Community-Based Interventions to Promote Increased Physical Activity. Appl. Health Econ. Health Policy.

[19] Vancampfort D., Hallgren M., Firth J., Rosenbaum S., Schuch F.B., Mugisha J., Probst M., Van Damme T., Carvalho A.F., Stubbs B. (2018). Physical Activity and Suicidal Ideation: A Systematic Review and Meta-Analysis. J. Affect. Disord..

[20] Schuch F.B., Vancampfort D., Firth J., Rosenbaum S., Ward P.B., Silva E.S., Hallgren M., Ponce De Leon A., Dunn A.L., Deslandes A.C. (2018). Physical Activity and Incident Depression: A Meta-Analysis of Prospective Cohort Studies. Am. J. Psychiatry.

[21] John A., McGregor J., Jones I., Lee S.C., Walters J.T.R., Owen M.J., O’Donovan M., DelPozo-Banos M., Berridge D., Lloyd K. (2018). Premature Mortality among People with Severe Mental Illness—New Evidence from Linked Primary Care Data. Schizophr. Res..

[22] Hoffmann K.D., Walnoha A., Sloan J., Buddadhumaruk P., Huang H.-H., Borrebach J., Cluss P.A., Burke J.G. (2015). Develop-ing a Community-Based Tailored Exercise Program for People With Severe and Persistent Mental Illness. Prog. Community Health Partnersh..

[23] Firth J., Rosenbaum S., Stubbs B., Gorczynski P., Yung A.R., Vancampfort D. (2016). Motivating Factors and Barriers towards Exercise in Severe Mental Illness: A Systematic Review and Meta-Analysis. Psychol. Med..

[24] Rebar A.L., Taylor A. (2017). Physical Activity and Mental Health; It Is More than Just a Prescription. Ment. Health Phys. Act..

[25] No Matter How Bad Things Are, We Can Help. https://www.rethink.org/.

[26] Kinnafick F.-E., Thøgersen-Ntoumani C., Duda J.L. (2014). Physical Activity Adoption to Adherence, Lapse, and Dropout: A Self-Determination Theory Perspective. Qual. Health Res..

[27] Lloyd-Evans B., Mayo-Wilson E., Harrison B., Istead H., Brown E., Pilling S., Johnson S., Kendall T. (2014). A Systematic Review and Meta-Analysis of Randomised Controlled Trials of Peer Support for People with Severe Mental Illness. BMC Psychiatry.

[28] Morrison P., Stomski N.J. (2015). Embracing Participation in Mental Health Research: Conducting Authentic Interviews. Qual. Res. J..

[29] Reardon T., Harvey K., Baranowska M., O’Brien D., Smith L., Creswell C. (2017). What Do Parents Perceive Are the Barriers and Facilitators to Accessing Psychological Treatment for Mental Health Problems in Children and Adolescents? A Systematic Review of Qualitative and Quantitative Studies. Eur. Child Adolesc. Psychiatry.

[30] Oates J. (2015). Use of Skype in Interviews: The Impact of the Medium in a Study of Mental Health Nurses. Nurse Res..

[31] Braun V., Clarke V. (2021). Can I Use TA? Should I Use TA? Should I Not Use TA? Comparing Reflexive Thematic Analysis and Other Pattern-Based Qualitative Analytic Approaches. Couns. Psychother. Res..

[32] Braun V., Clarke V. (2019). Reflecting on Reflexive Thematic Analysis. Qual. Res. Sport Exerc. Health.

[33] Braun V., Clarke V. (2014). What Can “Thematic Analysis” Offer Health and Wellbeing Researchers?. Int. J. Qual. Stud. Health Well-Being.

[34] Smith B., McGannon K.R. (2018). Developing Rigor in Qualitative Research: Problems and Opportunities within Sport and Exercise Psychology. Int. Rev. Sport Exerc. Psychol..

[35] Quirk H., Crank H., Harrop D., Hock E., Copeland R. (2017). Understanding the Experience of Initiating Community-Based Physical Activity and Social Support by People with Serious Mental Illness: A Systematic Review Using a Meta-Ethnographic Approach. Syst. Rev..

[36] Slade M., Longden E. (2015). Empirical Evidence about Recovery and Mental Health. BMC Psychiatry.

[37] Graham C.R., Larstone R., Griffiths B., de Leeuw S., Anderson L., Powell-Hellyer S., Long N. (2017). Development and Evalua-tion of Innovative Peer-Led Physical Activity Programs for Mental Health Service Users. J. Nerv. Ment. Dis..

[38] Malcolm E., Evans-Lacko S., Little K., Henderson C., Thornicroft G. (2013). The Impact of Exercise Projects to Promote Mental Wellbeing. J. Ment. Health.

[39] Smith B. (2018). Generalizability in Qualitative Research: Misunderstandings, Opportunities and Recommendations for the Sport and Exercise Sciences. Qual. Res. Sport Exerc. Health.

[40] Jetten J., Haslam C., von Hippel C., Bentley S.V., Cruwys T., Steffens N.K., Haslam S.A. (2022). “Let’s Get Physical”—or Social: The Role of Physical Activity versus Social Group Memberships in Predicting Depression and Anxiety over Time. J. Affect. Disord..

[41] Doré I., O’Loughlin J.L., Schnitzer M.E., Datta G.D., Fournier L. (2018). The Longitudinal Association between the Context of Physical Activity and Mental Health in Early Adulthood. Ment. Health Phys. Act..

