# Supplementary material for: Embedding Physical Activity into Community-Based Peer Support Groups for those Severely Affected by Mental Illness
Source: Int J Environ Res Public Health. 2023 Jan 27;20(3):2291. doi: 10.3390/ijerph20032291 (PMC9916288; doi:10.3390/ijerph20032291)
Supplement: Supplementary file 1 [file ijerph-20-02291-s001.zip › ijerph-2059799-supplementary.pdf]

Focus group and interview guide themes

<u>Peer support group member focus groups:</u>

1. Experience of peer support groups
2. Views on physical activity
3. Mental and physical health

<u>Peer support group leader interviews and focus groups:</u>

1. Views on physical activity
2. Process of embedding physical activity into peer support groups
3. Rethink Mental Illness toolkit
4. Impact of embedding physical activity into peer support groups
5. Impact of COVID-19 (for those conducted following the onset of the pandemic)

<u>Group Development Officers (GDOs) focus group:</u>

1. Overview of their role and responsibilities as GDOs
2. Engagement with group leads
3. Perceived availability of support for role
4. Motivation of groups (pre-post pandemic)
5. Types/format of activity used by groups (pre-post pandemic)
6. Lessons learnt

<u>Project Managers:</u>

1. Overview of their role and responsibilities as project managers
2. Reflection on the project pre-COVID-19
3. The importance of physical activity for the groups and the role of peer support
4. Impact of COVID-19 and changes required
5. The support offered to GDOs, Group Leaders and Group Members
6. Lessons learned and plans for the future
